# Prediction of Quadrupled Hamstring Graft Thickness for Anterior Cruciate Ligament Reconstruction Using Anthropometric Measurements: A Prospective Study

**DOI:** 10.7759/cureus.112714

**Published:** 2026-07-15

**Authors:** Amit Kumar, Kumar Shantanu, Devarshi Rastogi, Pratistha Srivastava, Brij Mohan Patel, Shatakshi Pant, Ashish Kumar

**Affiliations:** 1 Orthopaedic Surgery, King George's Medical University, Lucknow, IND; 2 Biochemistry, Santosh Deemed to be University, Ghaziabad, IND

**Keywords:** anterior cruciate ligament reconstruction, anthropometric parameters, graft diameter prediction, hamstring autograft, thigh circumference

## Abstract

Background: Anterior cruciate ligament (ACL) reconstruction using hamstring autograft is a standard procedure for restoring knee stability. Graft diameter is a key determinant of surgical success, with smaller grafts associated with higher failure rates. Preoperative prediction of hamstring graft size using simple anthropometric parameters may assist in surgical planning and improve outcomes.

Objectives: To evaluate the relationship between anthropometric measurements and quadrupled hamstring graft diameter in patients undergoing ACL reconstruction.

Methods: This prospective study was conducted at a tertiary care center over 18 months (August 2023-February 2025) and included 48 patients aged 18-55 years undergoing primary ACL reconstruction with hamstring autograft. Preoperative parameters recorded included age, gender, height, weight, body mass index (BMI), and thigh circumference. Graft diameter was measured intraoperatively using a graft sizing block. Statistical analysis was performed using IBM SPSS Statistics for Windows, Version 24 (Released 2016; IBM Corp., Armonk, New York), with p < 0.05 considered significant.

Results: The mean age was 40.5 ± 8.3 years, with male predominance (70.8%). Mean height, weight, and BMI were 165.7 ± 6.6 cm, 78.8 ± 7.9 kg, and 24.9 ± 3.9 kg/m², respectively. The mean thigh circumference and graft diameter were 53.0 ± 5.0 cm and 7.91 ± 0.82 mm. Height showed a strong positive correlation with graft diameter (r = 0.666, p < 0.001), while thigh circumference showed a moderate positive correlation (r = 0.367, p = 0.010). BMI demonstrated a significant negative correlation (r = -0.330, p = 0.022). Males had significantly larger graft diameters than females (8.30 ± 0.60 mm vs 6.96 ± 0.41 mm, p < 0.001).

Conclusion: Anthropometric parameters, particularly height, are reliable predictors of hamstring graft diameter in ACL reconstruction. Preoperative assessment can help identify patients at risk of inadequate graft size and guide surgical planning, including consideration of alternative graft options to optimize outcomes.

## Introduction

Anterior cruciate ligament (ACL) rupture represents a common and debilitating injury, particularly prevalent among athletes, leading to knee instability and significant long-term morbidity, including the potential for early-onset osteoarthritis [1]. Surgical reconstruction of the ACL has become the gold standard for restoring knee stability and function, with autografts, specifically those derived from hamstring tendons, being a frequently preferred choice due to their biomechanical properties and reduced donor site morbidity compared to patellar tendon grafts [2, 3]. However, a critical determinant of successful ACL reconstruction outcomes is the diameter of the harvested graft, with a quadrupled hamstring graft diameter of 7 mm or greater widely recognized as a benchmark for minimizing graft failure rates and ensuring long-term knee stability [2, 4-6]. Grafts smaller than this critical threshold are associated with a significantly higher risk of failure, recurrence of instability, and the need for revision surgery [2, 4].

The challenge in utilizing hamstring autografts lies in the inherent biological variability in tendon size among individuals, which makes precise preoperative prediction of graft thickness difficult [7]. This variability necessitates reliable methods for estimating graft size before surgery to allow for adequate surgical planning, including the potential need for alternative graft sources (e.g., allografts) or augmentation techniques (e.g., creating five-strand grafts or combining with synthetic materials) if the anticipated hamstring graft is insufficient [6, 8]. Accurate preoperative prediction is crucial for patient counseling, optimizing surgical efficiency, and ultimately improving patient outcomes [9].

Historically, researchers have explored the utility of simple anthropometric measurements as non-invasive, cost-effective tools to predict hamstring graft dimensions. These measurements typically include parameters such as height, weight, body mass index (BMI), age, gender, thigh circumference, and thigh length [4, 7, 10-12]. The rationale is that these macroscopic body parameters might correlate with the underlying size of hamstring tendons. Numerous prospective studies across diverse populations have investigated these correlations with varying degrees of success. For instance, studies have reported significant correlations between thigh circumference, height, and weight with hamstring graft thickness [10, 11]. However, the consistency and strength of these correlations have been inconsistent across different studies, influenced by factors such as ethnicity, geographical location, study methodology, and specific measurement protocols [2, 3]. For example, hamstring graft sizes have been shown to differ between Chinese and Caucasian populations, highlighting the need for population-specific predictive models [3].

Despite the appeal of anthropometric measurements for their simplicity, their predictive accuracy for individual graft thickness remains moderate, often limited by the complex biological variability of tendon architecture [13]. This has led to a growing emphasis on more direct methods of preoperative tendon assessment. Magnetic resonance imaging (MRI) has emerged as a promising tool, enabling direct visualization and measurement of semitendinosus and gracilis tendon cross-sectional areas (CSAs) prior to surgery. Several recent studies have demonstrated that MRI-based measurements can provide more accurate predictions of intraoperative graft size compared to anthropometric data alone, offering a more precise estimation of tendon dimensions [1, 6, 9, 13, 14]. Innovative approaches, such as using 3D tendon models from preoperative MRI, are also being explored to enhance predictive accuracy [1].

Therefore, the ongoing research into predicting quadrupled hamstring graft thickness represents a critical endeavor in orthopedic surgery. This field continues to evolve from relying primarily on anthropometric indicators to incorporating advanced imaging modalities. The ultimate aim is to develop robust, validated, and clinically applicable predictive models that integrate various patient-specific parameters to ensure optimal graft selection and improve the long-term success rates of ACL reconstruction.

## Materials and methods

This prospective study was conducted on patients undergoing primary ACL reconstruction at the Department of Orthopaedics, King George’s Medical University, Lucknow, over 18 months (August 2023-February 2025). A total of 48 patients aged 18-55 years with isolated ACL deficiency were included. Patients with prior ACL surgery, multiligament injuries, systemic diseases, or non-hamstring grafts were excluded.

Preoperative anthropometric parameters including age, gender, height, weight, BMI, and thigh circumference were recorded using standardized methods. Thigh circumference was measured 15 cm above the superior pole of the patella.

**Figure 1:**
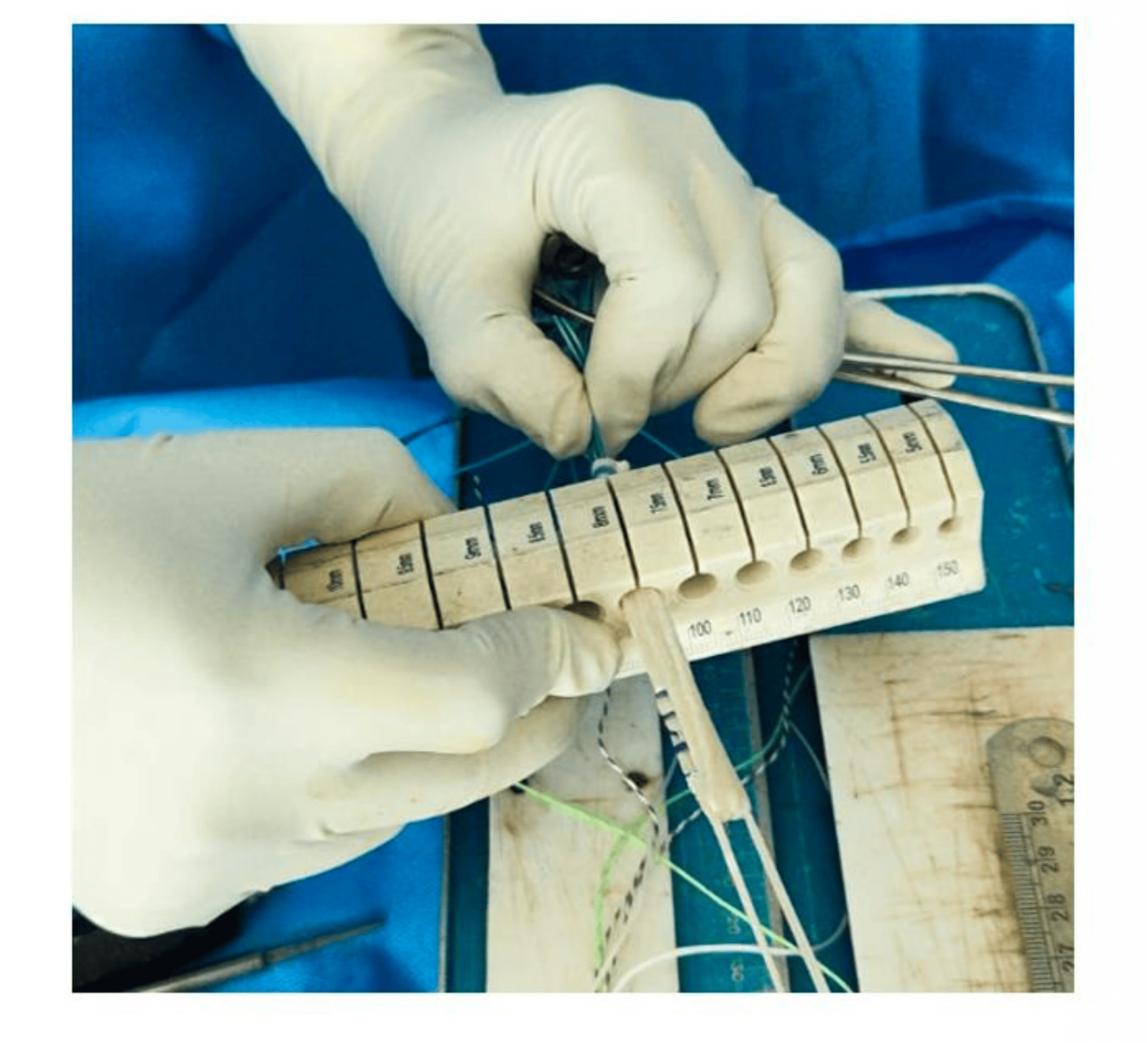
Intraoperative measurement of hamstring tendon graft diameter using a standardized graft-sizing block.

All surgeries were performed by a single senior surgeon under spinal anesthesia using a standardized arthroscopic technique. Semitendinosus and gracilis tendons were harvested through a small incision, prepared into a quadrupled graft, and the graft diameter was measured using a sizing block (Figure 1). Tibial and femoral tunnels were created anatomically, and graft fixation was achieved using an EndoButton on the femoral side and interference screw or cortical fixation on the tibial side.

The collected data were analyzed using IBM SPSS Statistics for Windows, Version 24 (Released 2016; IBM Corp., Armonk, New York). Continuous variables were summarized as mean ± standard deviation (SD), whereas categorical variables were presented as frequencies and percentages. To compare thigh circumference and hamstring graft diameter across different age groups, genders, and BMI categories, the independent samples t-test was used. The relationship between hamstring graft diameter and anthropometric parameters such as age, height, weight, BMI, and thigh circumference was evaluated using Pearson’s correlation analysis. A p-value of less than 0.05 was considered statistically significant for all analyses.

## Results

The age distribution of the 48 patients undergoing ACL reconstruction shows a mean age of 40.5 ± 8.3 years, with the majority (68.7%) falling within the 31-55-year age range. This demographic suggests that middle-aged individuals are more likely to undergo ACL reconstruction (Table 1).

**Table 1:** Baseline demographic and anthropometric characteristics of the study population (n = 48).

Variable	Category	Number of patients (n = 48)	Percentage (%)/mean ± SD
Age (years)	≤30	7	14.6%
	31-40	17	35.4%
	41-50	16	33.3%
	50-55	8	16.7%
	Mean ± SD	--	40.5 ± 8.3
Gender	Male	34	70.8%
	Female	14	29.2%
Height (cm)	--	--	165.7 ± 6.6
Weight (kg)	--	--	78.8 ± 7.9
BMI (kg/m²)	--	--	24.9 ± 3.9
BMI category	Normal (<25)	28	58.3%
	Overweight (25-29.9)	14	29.2%
	Obese (≥30)	6	12.5%

The gender distribution of the 48 patients (Table 1) undergoing ACL reconstruction reveals a male predominance, with males accounting for 70.8% (n = 34) of the cohort and females comprising 29.2% (n = 14). The anthropometric measurements of the 48 patients undergoing ACL reconstruction show a mean height of 165.7 ± 6.6 cm, weight of 78.8 ± 7.9 kg, and BMI of 24.9 ± 3.9 kg/m^2^. These values indicate that the average patient is of moderate height and weight, with a slightly elevated BMI. The BMI categorization of the 48 patients undergoing ACL reconstruction reveals that the majority (58.3%) have a normal BMI, while 29.2% are overweight and 12.5% are obese (Table 1).

The study's findings show a mean thigh circumference of 53.0 ± 5.0 cm and a mean hamstring graft diameter of 7.91 ± 0.82 mm. The range of thigh circumference (45.0-61.0 cm) and graft diameter (6.5-9.0 mm) suggests variability among patients. The study also found a significant difference in age group (less than 30 and more than 30 years) with thigh circumference (p = 0.037; Table 2).

**Table 2:** Comparison of demographic variables with thigh circumference and hamstring graft diameter (n = 48). Data are presented as mean ± standard deviation (SD). p-values were calculated using the independent samples t-test. *p < 0.05 (statistically significant); ***p < 0.001 (very highly significant. SD: standard deviation.

Variable	Category	Thigh circumference (cm) mean ± SD	p-value	Hamstring graft diameter (mm) mean ± SD	p-value
Age group	≤30 (n = 7)	56.1 ± 4.3	0.037*	7.7 ± 0.3	0.185
	>30 (n = 41)	52.51 ± 4.10		7.93 ± 0.88	
Gender	Male (n = 34)	54.2 ± 4.7	0.042*	8.30 ± 0.60	<0.001***
	Female (n = 14)	56.2 ± 3.1		6.96 ± 0.41	
BMI category	Normal (<25) (n = 28)	52.2 ± 4.6	0.043*	8.1 ± 0.75	0.038*
	Abnormal (>25) (n = 20)	55.24 ± 5.5		7.63 ± 0.85	

A total of 48 patients, including 34 males and 14 females, were compared for thigh circumference and hamstring graft diameter. Thigh circumference was significantly higher in females (56.2 ± 3.1 cm) than in males (54.2 ± 4.7 cm) with a p-value of 0.042 (significant). Hamstring graft diameter was significantly greater in males (8.30 ± 0.60 mm) compared to females (6.96 ± 0.41 mm), with a highly significant p-value of <0.001. The study found significant differences in thigh circumference and hamstring graft diameter across normal (BMI < 25) and abnormal (BMI > 25) BMI categories, with p-values of 0.043 and 0.038, respectively (Table 2).

**Figure 2:**
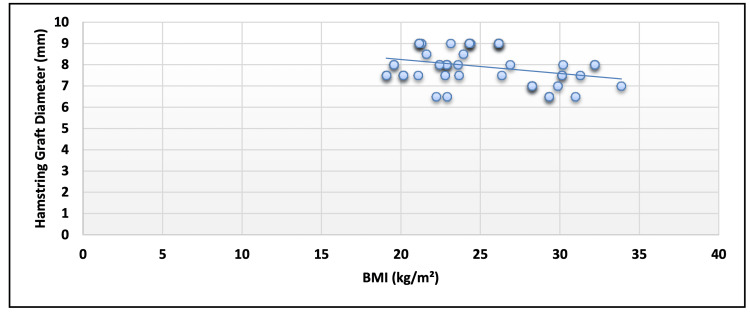
Correlation of hamstring graft diameter (mm) with BMI (kg/m²).

**Figure 3:**
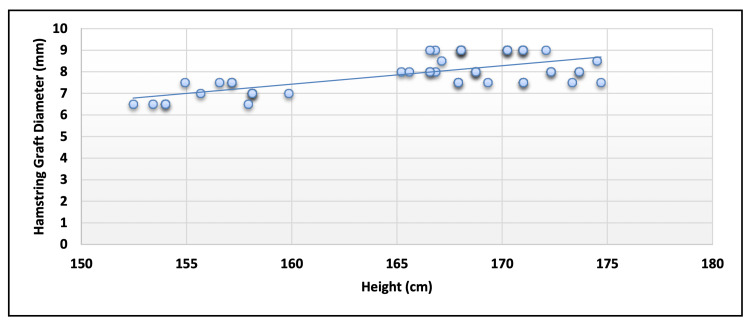
Correlation of hamstring graft diameter (mm) with height (cm).

**Figure 4:**
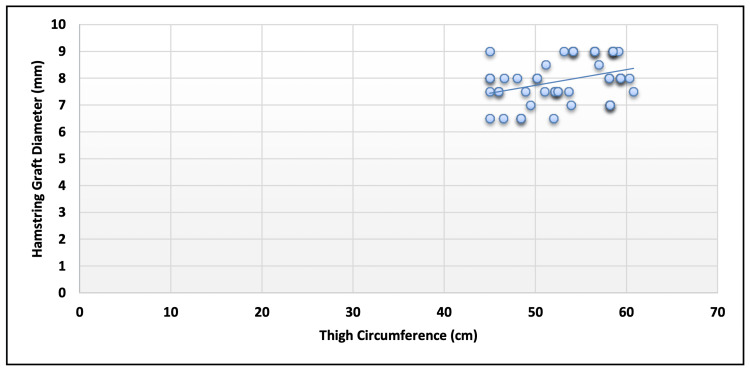
Correlation of hamstring graft diameter (mm) with thigh circumference (cm).

The Pearson correlation analysis reveals a significant negative correlation between hamstring graft diameter and BMI (Figure 2; r = -0.330, p = 0.022), indicating that individuals with higher BMI tend to have thinner grafts. Conversely, height (Figure 3; r = 0.666, p < 0.001) and thigh circumference (Figure 4; r = 0.367, p = 0.010) show a significant positive correlation with the hamstring graft diameter. This suggests that taller individuals and those with larger thigh circumference tend to have thicker hamstring grafts. Whereas no significant correlation of age and weight with hamstring graft diameter was observed (Table 3).

**Table 3:** Correlation analysis of hamstring graft diameter with clinical variables (n = 48). p-values were calculated using the Pearson correlation (r). *p < 0.05 (statistically significant); ***p < 0.001 (very highly significant).

Variable	r-value	p-value
Age (years)	-0.166	0.260
Height (cm)	0.666	<0.001***
Weight (kg)	-0.180	0.221
BMI (kg/m²)	-0.330	0.022*
Thigh circumference (cm)	0.367	0.010*

## Discussion

ACL reconstruction is a well-established procedure for restoring knee stability and function, with hamstring autografts being a commonly preferred option. Accurate preoperative prediction of graft thickness is essential for optimal surgical planning and decision-making.

In the present study, the mean age of patients was 40.5 ± 8.3 years, which is higher than that reported by Sakti et al. (27.23 ± 7.46 years), Asif et al. (29.4 years), Moghamis et al. (29 years), and Goyal et al. (30.2 years). This variation may be due to differences in inclusion criteria. However, age did not demonstrate a consistent correlation with graft diameter, consistent with previous literature [15, 3, 6, 2]. The mean height of patients was 165.7 ± 6.6 cm, comparable to Sakti et al. (167.76 ± 7.11 cm), Rinshad et al. (169.0 ± 7.1 cm), Asif et al. (172.6 cm), Moghamis et al. (174 cm), and Goyal et al. (169.1 ± 6.9 cm). Height showed a strong positive correlation with graft diameter (r = 0.666, p < 0.001), indicating that taller individuals tend to have thicker grafts [15-16, 3, 6, 2]. The mean weight was 78.8 ± 7.9 kg, slightly higher than Sakti et al. (71.9 ± 15.76 kg), Rinshad et al. (71.2 ± 10.4 kg), Asif et al. (70.9 kg), and Goyal et al. (72.2 kg), but comparable to Moghamis et al. (82.2 kg) [15-16, 3, 2, 6]. The mean BMI was 24.9 ± 3.9 kg/m², similar to Rinshad et al. (24.9 ± 3.3 kg/m²), slightly lower than Moghamis et al. (27.0 kg/m²) and Goyal et al. (25.6 kg/m²), and slightly higher than Asif et al. (23.8 kg/m²). BMI showed a weak negative correlation with graft diameter (r = −0.330, p = 0.022), suggesting limited predictive value [16, 6, 2, 3].

A statistically significant difference was observed across age groups (≤30 and >30 years) for thigh circumference (p = 0.037), whereas no significant difference was observed for hamstring graft diameter (p = 0.185). Gender-based differences revealed that females had a mean thigh circumference of 56.2 ± 3.1 cm and graft diameter of 6.96 ± 0.41 mm, whereas males had 54.2 ± 4.7 cm and 8.30 ± 0.60 mm, respectively (p < 0.05). This aligns with previous studies reporting larger graft sizes in males. When analyzed based on BMI categories (normal vs abnormal), significant differences were observed (p = 0.043 for thigh circumference and p = 0.038 for graft diameter), although overall BMI did not consistently predict graft size. Overall, correlation analysis confirmed that height and thigh circumference are significant predictors of hamstring graft diameter. These findings are consistent with previous studies, including Asif et al., who proposed the predictive equation: graft diameter (mm) = 0.079 × height (cm) + 0.068 × thigh circumference (cm) − 9.031, with strong predictive accuracy (R² = 0.8-0.9) [3].

The study highlights the importance of anthropometric measurements in preoperative planning, particularly in the Indian population, where such data are limited. Identifying patients at risk of a smaller graft diameter allows surgeons to consider alternative graft options and avoid intraoperative challenges. However, several limitations should be acknowledged. The study was conducted at a single tertiary care center and included a relatively small sample size, which may limit the generalizability of the findings. In addition, the study population consisted predominantly of male patients, which could have influenced the observed associations between anthropometric parameters and graft diameter. The inverse relationship observed between BMI category and thigh circumference should also be interpreted cautiously, as BMI does not distinguish between fat mass, lean muscle mass, or regional body composition. Only anthropometric variables were evaluated, while imaging-based predictors such as MRI-derived tendon CSA were not assessed. Furthermore, factors related to tendon quality and biomechanical properties, which may also affect graft size, were beyond the scope of the present study. Future multicenter studies with larger and more diverse populations, along with the incorporation of imaging-based assessments, are needed to validate and refine predictive models for hamstring graft diameter.

## Conclusions

This study demonstrates that anthropometric parameters play a significant role in predicting hamstring graft diameter in ACL reconstruction. Among the variables evaluated, height showed the strongest positive correlation with graft diameter, making it the most reliable predictor. Significant gender-based differences were also observed, with males having larger graft diameters than females. In addition, BMI demonstrated a negative correlation with graft diameter, suggesting that individuals with a higher BMI may have relatively smaller graft sizes.

Thigh circumference also showed a statistically significant positive correlation with graft diameter, although the strength of this association was weaker than that observed for height. These findings may help identify patients at risk of having smaller hamstring graft diameters and facilitate preoperative planning. Incorporating simple anthropometric measurements into routine preoperative assessment may aid graft selection, improve surgical decision-making, and enhance patient counseling in ACL reconstruction.
